# Supplementary material for: From sequence to enzyme mechanism using multi-label machine learning
Source: BMC Bioinformatics. 2014 May 19;15:150. doi: 10.1186/1471-2105-15-150 (PMC4229970; doi:10.1186/1471-2105-15-150)
Supplement: Additional file 2 — Java code of ml2db. Additional file ml2db_code.tar.gz contains the Java source code to run the multi-label machine learning experiments and save the results to database. The code’s Javadoc is included. [file 1471-2105-15-150-S2.zip › additional file 2/ml2db/utils/doc/edu/cornell/lassp/houle/RngPack/package-summary.html]

edu.cornell.lassp.houle.RngPack


JavaScript is disabled on your browser.


- Overview
- Package
- Class
- Use
- Tree
- Deprecated
- Index
- Help

- Prev Package
- Next Package

- Frames
- No Frames

- All Classes

# Package edu.cornell.lassp.houle.RngPack

- Class Summary

  | Class | Description |
  | --- | --- |
  |  |  |
  | --- | --- |
  | RandomApp | RandomApp is a simple application that demonstrates the use of RngPack. |
  | RandomElement | RandomElement is an abstract class that encapsulates uniform random number generators. |
  | RandomJava | RandomJava is a class wrapper for the `Math.random()` generator that comes with Java. |
  | RandomSeedable | `RandomSeedable` is an abstract class that extends the `RandomElement` class to include the ability to automatically generate a valid `long` seed from the clock. |
  | RandomShuffle | RandomShuffle uses one random number generator to shuffle the numbers produced by another to obliterate sequential correlations. |
  | Ranecu | Ranecu is an advanced multiplicative linear congruential random number generator with a period of aproximately 10^18^. |
  | Ranlux | RANLUX is an advanced pseudo-random number generator based on the RCARRY algorithm proposed in 1991 by Marsaglia and Zaman. |
  | Ranmar | RANMAR is a lagged Fibonacci generator proposed by Marsaglia and Zaman and is a good research grade generator. |

- Overview
- Package
- Class
- Use
- Tree
- Deprecated
- Index
- Help

- Prev Package
- Next Package

- Frames
- No Frames

- All Classes
